# Thinking forward: promising but unproven ideas for future intensive care

**DOI:** 10.1186/s13054-019-2462-1

**Published:** 2019-06-14

**Authors:** John J. Marini, Daniel DeBacker, Luciano Gattinoni, Can Ince, Ignacio Martin-Loeches, Pierre Singer, Mervyn Singer, Martin Westphal, Jean-Louis Vincent

**Affiliations:** 1 Erasmus University Medical Center, Rotterdam, Netherlands; 2 University of Muenster, Muenster, Germany; 3 CHIREC Hospitals, Brussels, Belgium; 4 University College London Medical School, London, UK; 5 Rabin Medical Center, Tel Aviv, Israel; 6 University of Gottingen, Gottingen, Germany; 7 St. James Hospital, Dublin, Ireland; 8 Erasme Hospital, Universite Libre de Bruxelles, Brussels, Belgium; 9 Regions Hospital, University of Minnesota, MS11203B, 640 Jackson Street, Minneapolis/St.Paul, MN 55101 USA

**Keywords:** Innovation, Paradigm, Ascorbic acid, Lactate, Nanotechnology, Post-intensive care syndrome, Nutritional support, Antibiotic overuse, Subcellular microscopy, Lithium

## Abstract

Progress toward determining the true worth of ongoing practices or value of recent innovations can be glacially slow when we insist on following the conventional stepwise scientific pathway. Moreover, a widely accepted but flawed conceptual paradigm often proves difficult to challenge, modify or reject. Yet, most experienced clinicians, educators and clinical scientists privately entertain untested ideas about how care could or should be improved, even if the supporting evidence base is currently thin or non-existent. This symposium encouraged experts to share such intriguing but unproven concepts, each based upon what the speaker considered a logical but unproven rationale. Such free interchange invited dialog that pointed toward new or neglected lines of research needed to improve care of the critically ill. In this summary of those presentations, a brief background outlines the rationale for each novel and deliberately provocative unconfirmed idea endorsed by the presenter.

## Introduction

The pathway to verified science often begins with intuition or flashes of insight. Without question, we need rigorous testing and confirmation before widespread implementation of a proposed innovation for medical practice. What seems to make conceptual sense is often proven wrong. In our field of critical care, however, it can be argued that what has become the traditional method of truth seeking has let us down. Attractive as it might seem, the clinical trial crucible is imperfect, inefficient, and applicable to a highly restricted number of testable questions. The list is long regarding initially accepted practices derived from the clinical trials base that later were overturned by more recent and more nuanced studies. Negative clinical trials tell us relatively little. Progress in our field, though undoubtedly real, has been rather slow and inefficient, prompting some experts to rethink the entire process of testing and validation. Yet, most experienced clinicians, educators, and clinical scientists privately entertain untested ideas about how care could or should be improved, even if the supporting evidence base is currently thin or non-existent. The following symposium encouraged each presenter to describe a personal favorite but unproven idea that might serve as a conceptual starting point along new or neglected lines of research or treatment aimed at improving care of the critically ill. We caution that although each presentation drew from well-established observations, the interpretations, extensions, and implications made by the presenters remain their own: speculative and unverified—at least for now.

### Unproven ideas for future critical care practice

#### Daniel De Backer: use of vitamin C as a therapeutic adjunct in sepsis

*Background*: While being around for some time for its anti-oxidant properties, vitamin C has been recently pushed under spotlight by a publication of Marik et al. suggesting that vitamin C, in addition to hydrocortisone and thiamine, may improve organ function and survival [1]. While this study has many limitations including its before/after design, this publication generated a lot of enthusiasm and several trials are nowadays underway trying to replicate these findings.

Vitamin C seems to have pleiotropic effects in sepsis. Vitamin C has been shown to increase arterial pressure in septic patients [1, 2], and this effect may be mediated by an increase in tetrahydrobiopterin availability, promoting catecholamine and vasopressin synthesis [2].

Vitamin C seems also to have beneficial effects on the microvasculature. In experimental sepsis, vitamin C improved microvascular perfusion. Interestingly, this protective microvascular effect remained effective even when vitamin C was administered up to 24 h after the onset of sepsis. This effect is dependent on endothelial NO synthase [3] and may be mediated by its impact on tetrahydrobiopterin, an essential cofactor of NO synthase. Interestingly, while promoting NO production on endothelial NO synthase, vitamin C also inhibits inducible NO synthase, limiting the production of detrimental radical species and in particular peroxynitrite.

However, even though vitamin C improved microvascular perfusion, it has limited impact on other markers of endothelial function. In particular, it failed to blunt the increase in endothelial permeability [4].

Hence, it seems that vitamin C may have interesting effects in sepsis, in particular through an improvement of endothelial reactivity and in microvascular perfusion. However, there remain many unsolved questions including adequate patient selection (should it be based on disease severity or based on vitamin c plasma levels?); timing of intervention (Just after initial resuscitation? At recognition of disease? As rescue therapy?); best dosing scheme, administered in isolation or combined with thiamine and hydrocortisone; and toxicity. These questions need to be solved in order to transform this unconfirmed idea into a clinical use.

*Idea*: Appropriately dosed vitamin C is a low-risk, low-cost, and potentially helpful addition to our current armamentarium used against sepsis and septic shock.

#### Luciano Gattinoni: impaired cellular O2 utilization, not sufficient perfusion and O2 availability, is the underlying defect causing “lactic acidosis” in most cases of septic shock

*Background*: Aggressive fluid resuscitation has become an accepted cornerstone of sepsis management, driven by the belief that hypotension and hyperlactatemia usually arise from inadequate oxygen availability in vital tissues [5]. However, hyperlactatemia may occur independently of tissue oxygen availability, as indicated by the coexistence of hyperlactatemia and low, normal, or high central venous oxygen saturation (ScvO_2_). In the absence of hypoxemia, central venous oxygen desaturation, although an imperfect marker, generally indicates greater than normal O2 extraction elicited by presumed inadequacy of O_2_ delivery. Yet, ScvO_2_ often remains normal in sepsis, even as metabolic acidosis proceeds. Moreover although the coexistence of hyperlactatemia and low pH is conventionally referred to as “lactic acidosis,” the direct link between excess lactate and acidemia is not straightforward [6]. In the traditional view, lactate and free protons [lactic acid] are the “abnormal” products of glycolysis in anoxic tissue. Yet, according to the “lactate shuttle theory,” lactate is the “normal” (i.e., expected) end-product of glycolysis, regardless of the oxygen tissue tension, is normally produced without a net release of free protons, and does not directly affect pH [7]. Thus, although lactate is a useful marker of the severity of shock [8], well-functioning kidneys regulate the electrolyte balance, compensating for the challenge to ionic balance, and acidemia seldom develops. In the absence of fully compensatory mechanisms, however, any excess of lactate must result in a negative base excess. In septic patients, such compensations may or may not be present, and therefore, there is no direct association between lactate levels and acidemia. Indeed, arterial pH, lactate, and ScvO_2_—which according to the traditional interpretative view should be tightly interlinked—are often present in contradictory combinations. Maintenance of normal ScvO_2_ suggests that targeting a further boost in oxygen *delivery* to peripheral tissues, e.g., by vigorous fluid administration, may be a misdirected response to lactic acidosis—especially when kidney perfusion and function are well preserved.

*Idea*: Modulation of the aggressiveness of fluid resuscitation in accordance with measurements of ScvO2 and determination of the base excess component not attributable to lactate—the “alactic base excess,” a simple calculation that could help assess renal compensating ability for the stress of metabolic acidosis.

#### Can Ince: functional imaging of living cells in vivo

**Fig. 1:**
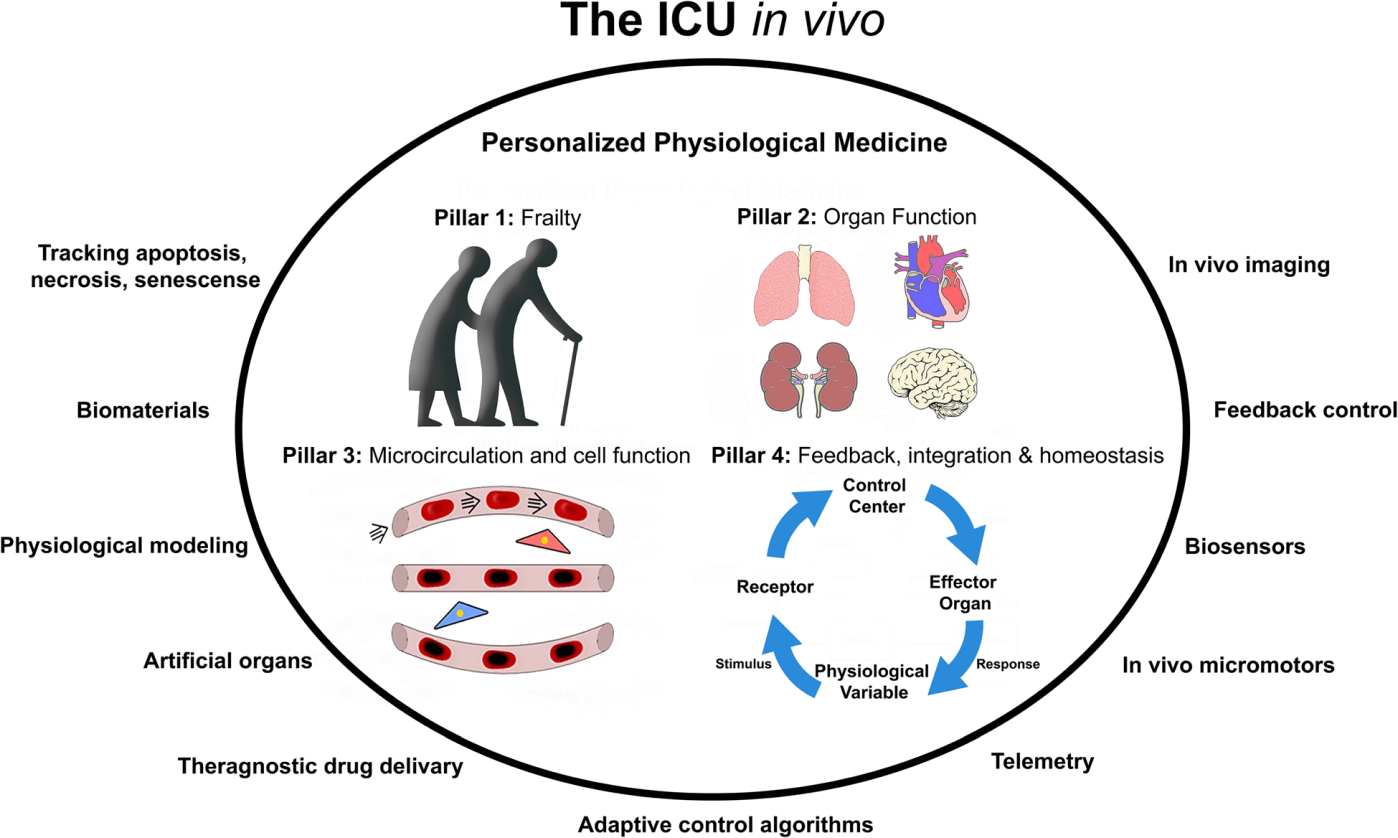
Plausible future tools for in vivo imaging, “theradiagnostics,” and precision drug delivery

*Background*: In recent years, remote imaging of the tubular structures of the gastrointestinal tract has been accomplished by radiofrequency communication from small ingested camera capsules. Although less invasive than traditional endoscopy, such methods simply establish the feasibility of acquiring anatomical data non-invasively at sites far removed from the caregiver. In the not so distant future, this principle will be extended to the cellular level within vasculature of key vital organs [9]. Perhaps more importantly, such imaging and tissue targeting may afford the opportunity to anatomically focus our interventions, such as drug delivery and assess their effects. Techniques already in development demonstrate the ability of synthetic biology devices to locate and tag vital tissues of interest [10]. Three currently being investigated are Caspase (apoptotic) tracking, specially engineered bacteria that home to tumor cells and tag by bioluminescence, and cell-type classifiers that determine key gene expressions and release marker molecules (Fig. 1).

Delivery of such trackers and of drugs may be actively driven by “micromotors” which are very small (micron-sized) particles that can move themselves [11]. These micromotors propel themselves in a specific direction autonomously when placed in a chemically appropriate liquid environment. Although potentially there are several different micromotor types and mechanisms applicable to medicine, key examples naturally occurring are the biological motors of bacteria and other self-propelled cells. Synthetically, researchers have exploited oxidation-reduction reactions to produce chemical gradients, local fluid flows, or streams of bubbles that propel these synthetic micromotors through the chemical media of body fluids. Methodologies to image their transit and that of other markers must be considered in the early stage of development.

Dynamic, contrast-enhanced ultrasound (CEUS) has been used experimentally to determine bubble transit time and thereby characterize perfusion through the microcirculation of the normal and sepsis altered kidney [12]. Other imaging methodologies that show considerable promise for bedside use include sidestream and incident darkfield imaging hand-held vital microscopes [13]. Such hand-held vital microscopes have been shown capable of imaging the richness and filling of microvessels and in identifying and quantifying leukocytes in the microcirculation of accessible tissues (e.g., sublingual) in a variety of non-invasive and surgical settings [14]. Although progress has been rapid, to this point, tissues substantially deeper than the subsurface layer remain out of reach by these methodologies. The time does not seem so far off that precision medicine will be abetted by intravascular telemetric nano-imaging, self-propelled searching capsules, in vivo functional assessment of targeted cell function, and the imaging of the benefit and consequences of drug delivery.

*Idea*: Microcirculatory imaging, cellular function assessment, and tissue-specific therapeutic targeting by nano-biotechnology.

#### John J. Marini: cognitive decline during and following ICU care may be averted by encouraging cues that maintain normal variations of physiology and behavior

*Background*: As often said, “variety is the spice of life.” More than that, variations of physiology, alertness, and cognition are essential components and markers of health; loss of such variations characterizes depleted reserves and serious, life-threatening illness [15]. For example, variations of respiratory breathing depth and rhythm exemplify effortless breathing, whereas the stress of exercise narrows that variation to a more metronome-like pattern. Narrowing of heart rhythm variability has been linked to loss of cardiac reserve. Circadian rhythms of many organ systems, e.g., certain key stress hormones, e.g., cortisol, also blunted by critical illness and are not restored by such uni-modal ICU interventions as light variation [16]. In the throes of the initial crisis, the body responds to the challenge by exuberant and often dysfunctional responses. As clinicians, we seek to blunt those responses and to “stabilize” physiologic parameters. Understandably, our initial goal is to achieve a viable plateau and to minimize variations from our set standard. We provide monotonous tidal volumes, fluid infusions, vasopressors, and feedings. We suppress alertness, consciousness, and pain. These interventions, while appropriate for the initial stage, have the well-recognized potential for harm when inappropriately prolonged [15].

The post-intensive care syndrome (PICS) is a pervasive and highly morbid consequence of critical illness and its management. Current approaches to its prevention focus on early mobilization, attention to avoiding excessive sedation, preventing delirium [17], and preserving sleep quality. These help preserve muscle strength and in the prevention and treatment of delirium [18]. Recent trials of non-pharmacologic interventions, however, have yielded disappointing results [19]. No approach to date has been shown to halt PICS-associated cognitive decline.

Another common adage is that “what you do not use, you lose.” Myriad physical and psychosocial signals normally keep the healthy brain oriented to the variability of daily life, and functional reinforcement and pruning of synaptic pathways may be occurring continually, especially during sleep [20]. Extraction of the patient from the familiar to the unfamiliar, threatening, and monotonous routines of the ICU that that normal environment may be adversely train the brain, forming dysfunctional connections and pruning away many that are normally used. It is also possible that monotonous two-way crosstalk between body and brain may deliver neuronal messages of ongoing disease, rather than of recovering health and resilience. Finally, as sustained isolation of even the healthy mind suggests, delirium might be the “default state” to which we might all regress if removed from re-orienting cues of daily living.

*Idea*: We should prioritize maintenance of normal daily psychosocial interactions and variations of activity during the post-resuscitation and recovery phases of ICU care to help prevent cognitive decline and debility.

#### Ignacio Martin-Loeches: highly personalized medicine to optimize sepsis management and outcomes

*Background*: At present, sepsis remains a potentially lethal condition approached from the platform of imprecise working diagnosis. Some patients benefit from this style of management, whereas many others receive little benefit or are even harmed by our attempts to help. Although somewhat controversial, a significant body of evidence indicates that early administration of appropriate antibiotics aids in achieving a satisfactory result. Currently, the two components of this strategy, appropriate antibiotics and early intervention, are both suboptimal. Lacking accurate microbiological diagnosis at treatment onset, powerful broad spectrum antibiotics with unwanted side effects are typically given that are later pared and/or replaced once the organism and sensitivities are known. In a significant number of cases, the responsible organism responsible for sepsis—if any—remains unknown. Likewise, we have made clear progress toward earlier antimicrobial intervention, but systemic logistical delays between order and “hang time” occur all too commonly along the “supply chain” of critical care delivery. Currently deployed biomarkers such as procalcitonin and c-reactive protein help establish and quantitate the intensity of tissue injury response but do not nail the responsible organism.

The response of the host to the septic challenge is a key and largely under-supported determinant of outcome. Some patients have an overly exuberant defensive response to the infective challenge that extends the attack to healthy tissues (e.g., vasoplegia and ARDS), whereas others are constitutively or functionally immunosuppressed and need a boost. For example, hydrocortisone may benefit those with plasma cytokine levels above a certain threshold, whereas others are not helped (or even injured) by steroid therapy [21]. On the other hand, polyclonal antibodies to boost immunity may benefit only those whose concentrations of immunoglobulins and inflammatory markers are low and high, respectively. Even when such evaluations can be implemented, not all septic patients will benefit; the degree of organ dysfunction, as indicated by the SOFA score, may be a helpful determinant of responsiveness.

A variety of well-established and newer tools for improving diagnostic and therapeutic precision are on the near horizon [22]. Nanotechnologies hold promise to quickly diagnose, selectively treat, and monitor the root causes of these septic conditions [23]. Apart from improved precision of antibiotic targeting, other novel treatments have shown exciting potential. For example, in well-selected patients, exogenous mesenchymal stem cells (MSCs) may help treat sepsis by their intrinsic ability to home to injured tissue, secrete paracrine signals to limit systemic and local inflammation, decrease apoptosis in threatened tissues, stimulate neoangiogenesis, activate resident stem cells, beneficially modulate immune cells, and exhibit direct antimicrobial activity. These effects are associated with reduced organ dysfunction and improved survival in animal models. Finally, agents such as cilengitide may prevent adherence of pathogens such as *Staphylococcus aureus* and *Escherichia coli* to the blood vessel endothelium, with experimentally demonstrated therapeutic benefit [24].

*Idea*: Utilize newer precision tools to rapidly identify the underlying causes, select well-targeted treatments, and boost host responses to sepsis.

#### Mervyn Singer: restricted and abbreviated use of antimicrobials for infection

*Background*: Few therapies are as well entrenched in ICU practice as the early and aggressive use of antibiotics to suppress infection [25]. A well-discussed and appropriate concern is the need to avoid the indiscriminate use of these agents to prevent emergence of life-threatening microbial resistance to these vital armaments. Yet, the idea that appropriate antibiotics are invariably helpful when infection develops is never seriously challenged. It may be wise to re-examine this mandate. Antibiotics are effective not only against the offending organism but also the host tissues, as well. Apart from their widely acknowledged potential for side effects, renal and hepatic dysfunction, the ability of certain agents to impair mitochondrial function (e.g., linezolid) [26] and to adversely alter both immunity and the microbiome is extensively documented. Few would withhold an effective antimicrobial in the face of a known symptomatic infection. However, considering their predictable tendencies to produce adverse consequences, one might logically wonder: “Just how solid is the evidence that antibiotics reduce mortality?” It turns out, less reassuring than one might think.

Many patients survived what might be considered life-threatening illnesses in the pre-antibiotic era. Early comparisons of sulphonamide antibiotics with placebo demonstrated a modest reduction of mortality from pneumonia, but an increased incidence of complications (e.g., empyema). In the pre-antibiotic eras that preceded our own, surgery of chest and abdomen for advanced infections was frequently successful without their use. In more recent years, enthusiasm for earliest possible antibiotic intervention has waned with the demonstration by multiple prospective studies that reasonable delays make very little difference to eventual outcome of sepsis and septic shock [25, 27]. Some experimental data indicate that the rapid early release of bacterial cell products by bactericidal antibiotics exacerbate severity. In certain settings, biomarker-targeted decisions for antibiotic intervention rather than automatic broad spectrum coverage may often make better sense [28], and de-escalation strategies for life-threatening infections appear to offer a survival advantage over sustained antibiotic treatment. Very recent reports indicate that fewer antibiotics and shorter treatment courses lessen adverse side effects and might even improve survival. Indeed, courses as brief as 1–3 days may be as effective as those traditionally recommended [29]. Taken together, such data prompt careful consideration of the need for antibiotic treatment and urge that targeted antibiotics be given for the shortest effective time.

*Idea*: Curtail the duration of antimicrobial treatment and avoid the use of antibiotics whenever feasible to do so.

#### Pierre Singer: precision nutrition for intensive care

*Background*: The sustained stresses of critical illness require appropriate and individualized nutritional support but often outstrip our current attempts to provide it. These shortcomings undoubtedly contribute to deficits that contribute to lasting disability that is both difficult and slow to repair. Our current approaches are rather indiscriminate, fail to scale appropriately to individual needs, and are unmodified during the changing requirements during the rescue, recovery, and re-strengthening phases of the patient’s ICU trajectory. Deficits of calories and protein develop quickly and early in the hospital course, deepening as the relatively immotile gut is unable to accept full target loads by the enteral route, often resulting in regurgitation or interruption of enteral administration. Moreover, the gastrointestinal tract may not absorb with normal efficiency (due to edema, etc.) and insufficient energy is delivered to cope with the metabolic stress. The natural pattern of intermittent oral meals almost invariably yields to continuous feedings with monitoring of gastric residuals—is nutritional support provided at all during the initial demanding phase? Finally, assessment of nutritional balance—requirements and demands—is currently confined to relatively crude and/or slowly reactive measurements of serum proteins such as albumin and its precursors, electrolytes, and glucose.

There has been substantial progress along multiple fronts to address these shortcomings [30]. Among many advances, the gut microbiome perhaps has received the most attention for its value in determining and assessing patient health status. Although of intense interest, this element of nutritional science does not stand alone. Smart nasogastric tubes that sense and maintain appropriate gut positioning and nutrient infusion rates (so as to avoid intolerance, regurgitation and vomiting) should help reach nutritional goals and avoid both discomfort and complications.

Improved understanding and recent innovations in technology and informatics that are applicable to the bedside promise to advance the precision and appropriateness of our future nutritional care. For example, the quantity of exhaled carbon dioxide can be noninvasively measure on an ongoing basis to give a clearer picture of the energy needs. The burgeoning fields of metabolomics and proteomics hold the potential to assess nutritional status, set appropriate adjustments of the nutrient mix, and monitor progress through the varied stages of acute illness. Recent evidence indicates that such enhanced precision may be profitably integrated into severity of illness indexes such as the SOFA score, cluster analyses, and inflammatory cytokine marker profiles to prognosticate with enhanced proficiency the outcome of life-threatening sepsis [31]. “Big Data” analytics of relevant populations and perhaps of the myriad points of physiologic data and laboratory variables that pertain to the individual have only begun to show their decision support potential [32].

*Idea*: Utilize modern advances in technological, molecular science, and informatics to better monitor and deliver nutritional requirements for optimized and nuanced care for the individual through all stages of critical illness.

#### Martin Westphal: lithium to treat disorders of the “bipolar” kidney

*Background*: Lithium is a very reactive metal representing a key constituent of powerful modern batteries including electric cars. Its clinical use goes back to 1847, when Dr. Alfred Garrod investigated the role of lithium in the treatment of gout. Interestingly, this approach was successful only in patients suffering from bipolar disorders as co-morbidity. However, it took more than 100 years to launch lithium as mood stabilizer in patients with bipolar disorders and to leverage its effects for the treatment of mania and prophylaxis of manic-depressive illness (http://www.lowdoselithium.com/the-history).

Recent research in rodent models revealed that lithium also exerts meaningful neuroprotection in stroke, fragile X syndrome, amyotrophic lateral sclerosis, and Huntington’s, Alzheimer’s, and Parkinson’s disease [33]. Among others, inhibition of glycogen synthase kinase type 3 [GSK3] appears to play a key role in this context. GSK3 is a ubiquitously expressed serine/threonine protein kinase, implicated in the processes of tissue injury, repair, and regeneration in multiple organ systems. Likewise, there is evidence that inhibition of this protein kinase conveys renal protection [34].

In fact, lithium inhibits GSK3, thereby blunting inflammation and oxidative stress as well as mediating kidney tissue repair [35]. It has been shown in a murine model of lipopolysaccharide [LPS]-elicited acute kidney injury (AKI) that a single-intraperitoneal injection of a low dose of lithium chloride [40 mg/kg] at the time of LPS administration reduces renal tissue injury, inflammation, and dysfunction, which is associated with a survival benefit [36]. Likewise, it has been reported that a delayed administration of lithium attenuates cisplatin- and ischemia/reperfusion-induced AKI in a dose-dependent manner. Specifically, lithium promotes renal tubular epithelia repopulation, improves kidney repair, and accelerates renal salvage. The authors concluded that pharmacologic targeting of GSK3 represents a promising approach to treat established AKI [37].

Currently, lithium is registered neither for prevention nor for treatment of AKI, but clinical studies are underway to elucidate its efficacy. Since high doses of lithium have been linked to kidney damage in the past [33], a better understanding of the dose-response relationship is crucial from a safety point of view. Once the safety is established, re-purposing lithium with the goal to prevent and treat AKI is straightforward and could make the depressed kidney happy.

*Idea*: Low-dose lithium may represent a viable option both to prevent AKI in patients at risk and to treat established AKI.
